# Neural differentiation of canine mesenchymal stem cells/multipotent mesenchymal stromal cells

**DOI:** 10.1186/s12917-020-02493-2

**Published:** 2020-08-10

**Authors:** Sonja Prpar Mihevc, Vesna Kokondoska Grgich, Andreja Nataša Kopitar, Luka Mohorič, Gregor Majdič

**Affiliations:** 1 Veterinary Faculty, Institute of Preclinical Sciences, University of Ljubljana, Gerbičeva 60, 1000 Ljubljana, Slovenia; 2 Faculty of Medicine, Institute of Microbiology and Immunology, University of Ljubljana, Zaloška 4, 1000 Ljubljana, Slovenia; 3 Animacel Ltd, Mivka 34, 1000 Ljubljana, Slovenia

**Keywords:** multipotent mesenchymal stromal cells, mesenchymal stem cells, dog, differentiation, neurons, astrocytes

## Abstract

**Background:**

The ability of adipose tissue-derived multipotent mesenchymal stromal cells/mesenchymal stem cells (ASCs) to differentiate in neural lineages promises progress in the field of regenerative medicine, especially for replacing neuronal tissue damaged by different neurological disorders. Reprogramming of ASCs can be induced by the growth medium with neurogenic inductors and specific growth factors. We investigated the neural differentiation potential of canine ASCs using several growth media (KEM, NIMa, NIMb, NIMc) containing various combinations of neurogenic inductors: B27 supplement, valproic acid, forskolin, N2-supplement, and retinoic acid. Cells were first preconditioned in the pre-differentiation neural induction medium (mitogenically stimulated; STIM1), followed by the induction of neuronal differentiation.

**Results:**

After 3, 6, and 9 days of neural induction, elongated neural-like cells with bipolar elongations were observed, and some oval cells with light nuclei appeared. The expression of neuronal markers tubulin beta III (TUBB3), neurofilament H (NF-H), microtubule-associated protein-2 (MAP2), and glial fibrillary acidic protein (GFAP) was observed using immunocytochemistry, which confirmed the differentiation into neurons and glial cells. Flow cytometry analysis showed high GFAP expression (between 70 and 90% of all cells) after cells had been growing three days in the neural induction medium a (NIMa). Around 25% of all cells also expressed adult neuronal markers NF-H and MAP2. After nine days of ASCs differentiation, the expression of all neural markers was reduced. There were no differences between the neural differentiation of ASCs isolated from female or male dogs.

**Conclusions:**

The differentiation repertoire of canine ASCs extends beyond mesodermal lineages. Using a defined neural induction medium, the canine ASCs differentiated into neural lineages and expressed markers of neuronal and glial cells, and also displayed the typical neuronal morphology. Differentiated ASCs can thus be a source of neural cellular lineages for the regenerative therapy of nerve damage and could be useful in the future for therapy or the modelling of neurodegenerative diseases.

## Background

Multipotent mesenchymal stromal cells (MSCs), also commonly referred to as mesenchymal stem cells are self-renewing, multipotent, adult stem cells that have a mesodermal and neuroectodermal origin [1, 2]. They are found in many tissues, such as adipose tissue, bone marrow, cord blood, chorionic folds of the placenta, amniotic fluid, blood, lungs, etc., most of which are easily accessible and represent a potentially significant source of cells. The ability of MSCs to differentiate into osteoblasts, chondroblasts, and adipocytes in *in vitro* conditions has been demonstrated in numerous studies [3, 4]. Until the year 2000, a widely accepted hypothesis stated that MSCs are capable of differentiating only into mesodermal lineages. However, this was challenged when rat MSCs, isolated from the bone marrow and exposed to butyl hydroxyanisole, β-mercaptoethanol, and dimethylsulfoxide started to express proteins specific to the nervous system [5].

Most studies on neural differentiation of MSCs were carried out with human and rodent cells [2, 5–13]. In veterinary medicine, dogs are interesting for the development of novel regenerative treatments, and in addition to benefiting canine patients, these therapies might show translational potential as dogs could be a highly interesting model of human neurological disorders. A few studies have reported the induction of canine MSCs into neural lineages [14–17], but there is no standard and optimized protocol for the neuronal induction of canine MSCs. GFAP, MAP2, A2B5, S100, TUBB3, nestin, and NEUN are markers of neural cells and can be used as markers of cellular differentiation *in vitro*. Canine adipose tissue-derived multipotent mesenchymal stromal cells (ASCs) could be induced to express some of these neuronal genes after growing in the presence of the neurogenic inductors valproic acid and forskolin [14, 16], basic fibroblast growth factor (bFGF), and epidermal growth factor (EGF) [15], in a commercial neurogenic differentiation medium [18] and in a medium containing N2 supplement, brain-derived neurotrophic factor (BDNF), and nerve growth factor (NGF) [19]. Neurospheres generated from the canine ASCs were used to enrich for neural lineages sufficiently expanded to transplant [19] and, when grown in hypoxia, showed higher expression of the neuronal marker nestin [20]. Recently, the neural differentiation of human ASCs was induced by a conditioned medium obtained from glial cells [21].

In the present study, four different cell growth media were tested to determine their ability to induce differentiation of canine ASCs into neural lineages. The media components included B27 supplement, valproic acid, forskolin, N2-supplement, and retinoic acid. One of the tested media enabled prominent neural differentiation, observed by morphological changes at the cellular level, as well as by the expression of glial marker GFAP and neuronal markers NF-H and MAP2, detected by immunocytochemical staining and flow cytometry.

## Results

### Morphology

**Fig. 1:**
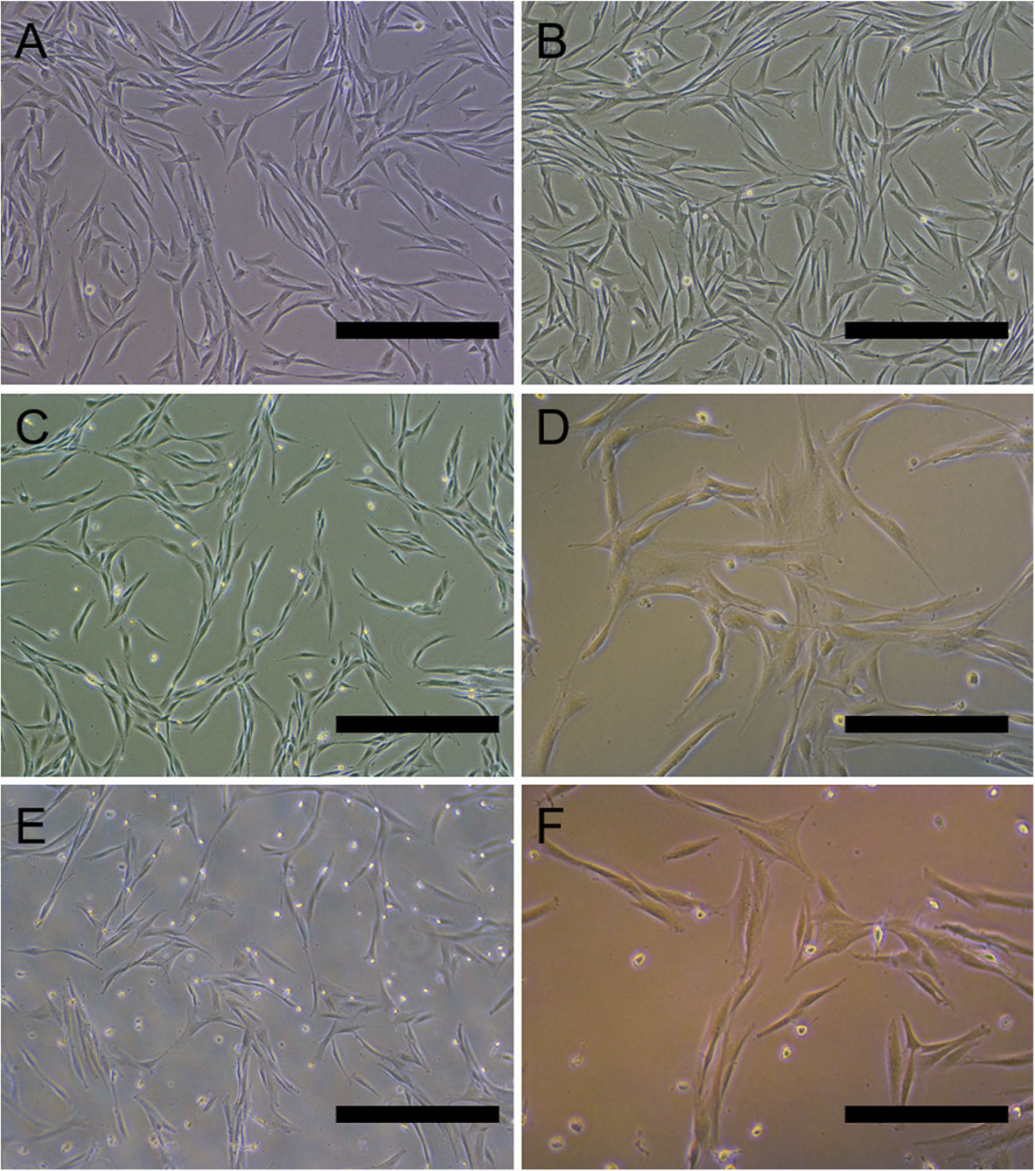
Canine ASCs typical growth pattern prior to differentiation (**a**, **b**) and their cellular morphology after 24 h (**c**, **d**) and 48 h (**e**, **f**) in pre-differentiation medium STIM1. Scale bars are 200 µm (**a**, **b**,** c**,** e**) and 100 µm (**d**, **f**)

The morphological characterization of ASCs was assessed at 6 time points: untreated cells at 80% confluence, 24 and 48 h after addition of pre-differentiation medium and 3, 6, and 9 days after induction of the differentiation of ASCs. Canine ASCs cultured in basal growth medium displayed typical fibroblast-like morphology (Fig. 1a and b).

Pre-differentiation medium STIM2 was highly toxic to the cells, while medium STIM1 was suitable for cell culture. STIM1 contained L-glutamine, B27 supplement, and two growth factors: EGF and bFGF. The pre-differentiation was introduced in order to enhance the proliferation of ASCs (by EGF and bFGF) and to slowly direct the lineage commitment to neuronal cell types. Neural cells were maintained in this medium by B27 supplement, which includes retinoic acid. The first morphological changes were observed after 24 h of treating ASCs with the pre-differentiation medium STIM1 when neuronal-like cells appeared, identified by their elongated shape with bipolar elongation (Fig. 1c and d). Additionally, some oval cells with light nuclei also appeared (Fig. 1c). The same cellular morphology was observed after 48 h of growth in the STIM1 medium, but more dead cells were detected (Fig. 1e and f). ASCs seeding density affected the efficiency of differentiation, which suggests that cell communication is essential for differentiation of ASCs into neural phenotypes.

**Fig. 2:**
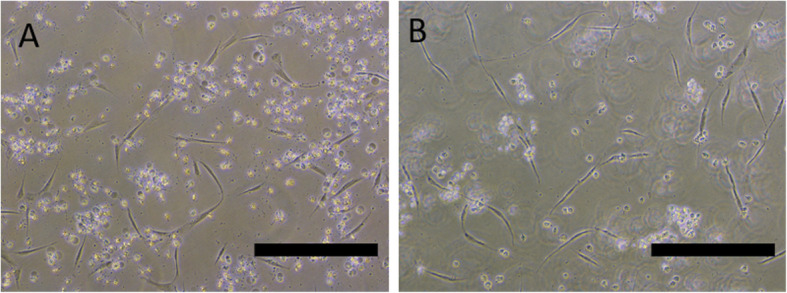
Canine ASCs grown in KEM differentiation medium for 1 (**a**) and 3 days (**b**). Prior to the addition of KEM, cells were grown in pre-differentiation medium STIM1 for 24 h. Scale bars are 200 µm

After pre-differentiation, cells were grown in four different differentiation media. The KEM medium was highly toxic to cells, which died after 3 days in this medium (Fig. 2). Its toxic effects were probably due to the abundance of neural differentiation-inducing factors; therefore, we further tested only three differentiation media with different compositions.

**Fig. 3:**
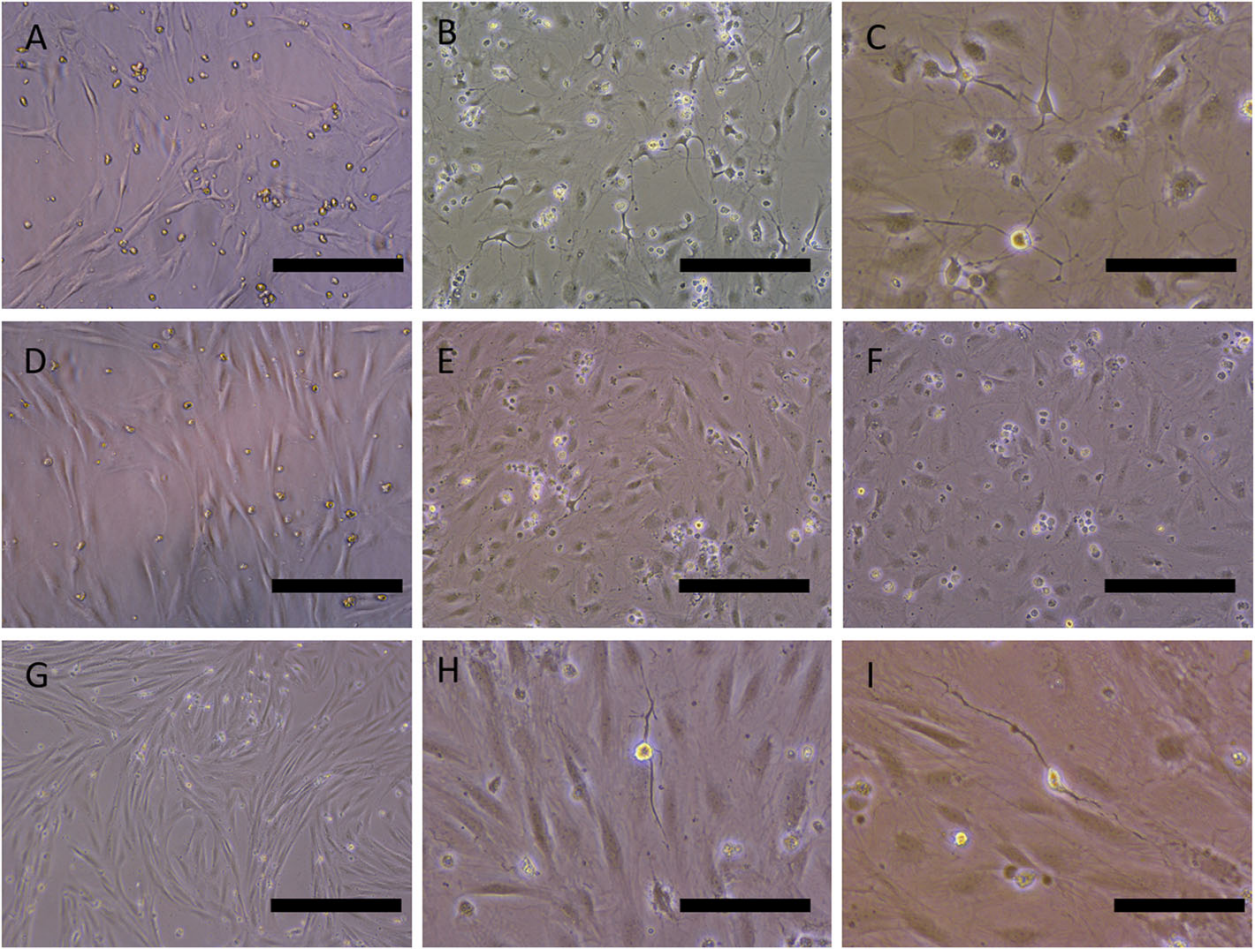
Morphology of canine ASCs grown in NIMa (**a**, **b**, **c**), NIMb (**d**, **e**, **f**) or NIMc (**g**, **h**, **i**) differentiation media for 3 (**a**, **d**, **g)**, 6 (**b**, **e**, **h**) or 9 (**c**, **f**, **i**) days. Cells were first exposed to pre-differentiation medium STIM1 for 24 h. Numerous thin protrusions resembling axons or dendrites are clearly seen in Figures **b** and **c**. Scale bars are 100 µm (**a**, **c**, **h**, **i**) and 200 µm in all other images

NIMa, NIMb, and NIMc all contained L-glutamine, B27, and N2 supplements and varying concentrations of retinoic acid. During growth in the NIM differentiation media (Fig. 3), the number of cells with neuronal phenotype increased, and they appeared more branched. At 3 days after the onset of differentiation, some cells obtained a neuron- or glial-like morphology with tree-like processes (Fig. 3a, d, and g). The concentration of retinoic acid in NIMa, NIMb, and NIMc media was 10 nM, 100 nM, and 10 µM, respectively. The most prominent neural-like morphologic changes were observed after 6 days of cultivation in the NIMa medium (Fig. 3b), although they were also observed in the other two media. The concentration of retinoic acid in NIMb and NIMc might have been too high, since many apoptotic cells were also observed. Therefore, the NIMa medium was determined to be the most suitable neural induction medium and all subsequent experiments were conducted with it. Following 9 days of incubation in the NIMa medium, neural-like cells appeared. These cells were more elongated and branched, and their cytoplasmic elongations resembled dendrites (Fig. 3c).

### Viability

**Fig. 4:**
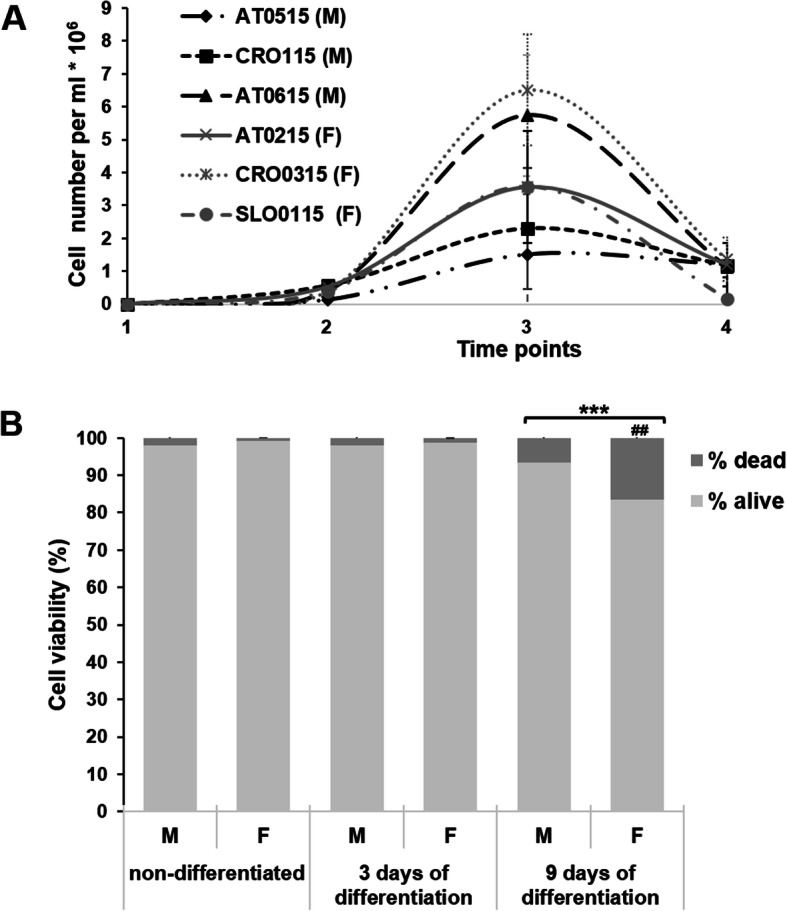
Growth curves (**a**) and viability plots (**b**) for ASCs. **a** Growth curves for six canine ASCs before and after the induction of differentiation. At time point 1, cells were seeded and time point 2 represents the number of cells at the beginning of the treatment with pre-differentiation medium STIM1, which was applied for 24 h. Cell numbers increased after 3 days of differentiation (time point 3) but decreased again after 9 days of differentiation (time point 4), although differences in cell numbers were not statistically significant. **b** Average viability of male and female dogs derived ASCs before and after the induction of differentiation. The viability decreased in both male and female cells on day 9 of cell culture (^***^P < 0.001), and there was statistically significant difference in viability between male and female cells (^##^*P* < 0.01) at this time point

Cell numbers were determined at three time points; 10^5^ cells were seeded per ml of growth medium, and cells were counted after 24 h of pre-differentiation in the STIM1 medium and at 3 and 9 days after induction of differentiation by the NIMa medium (Fig. 4a). No differences in growth dynamics between cells derived from male and female dogs were observed at 24 and 72 h after seeding (Fig. 4a). However, the viability of cells was reduced on the ninth day of differentiation, and this reduction was statistically significant (*p* < 0.001). Interestingly, the viability on the ninth day of cell culture was significantly lower with cells from female donors in comparison to cells from male dogs, i.e., 83.4 ± 1.0% vs 93.5 ± 2.3%, respectively (*p* < 0.01) (Fig. 4b).

### Immunofluorescence

**Fig. 5:**
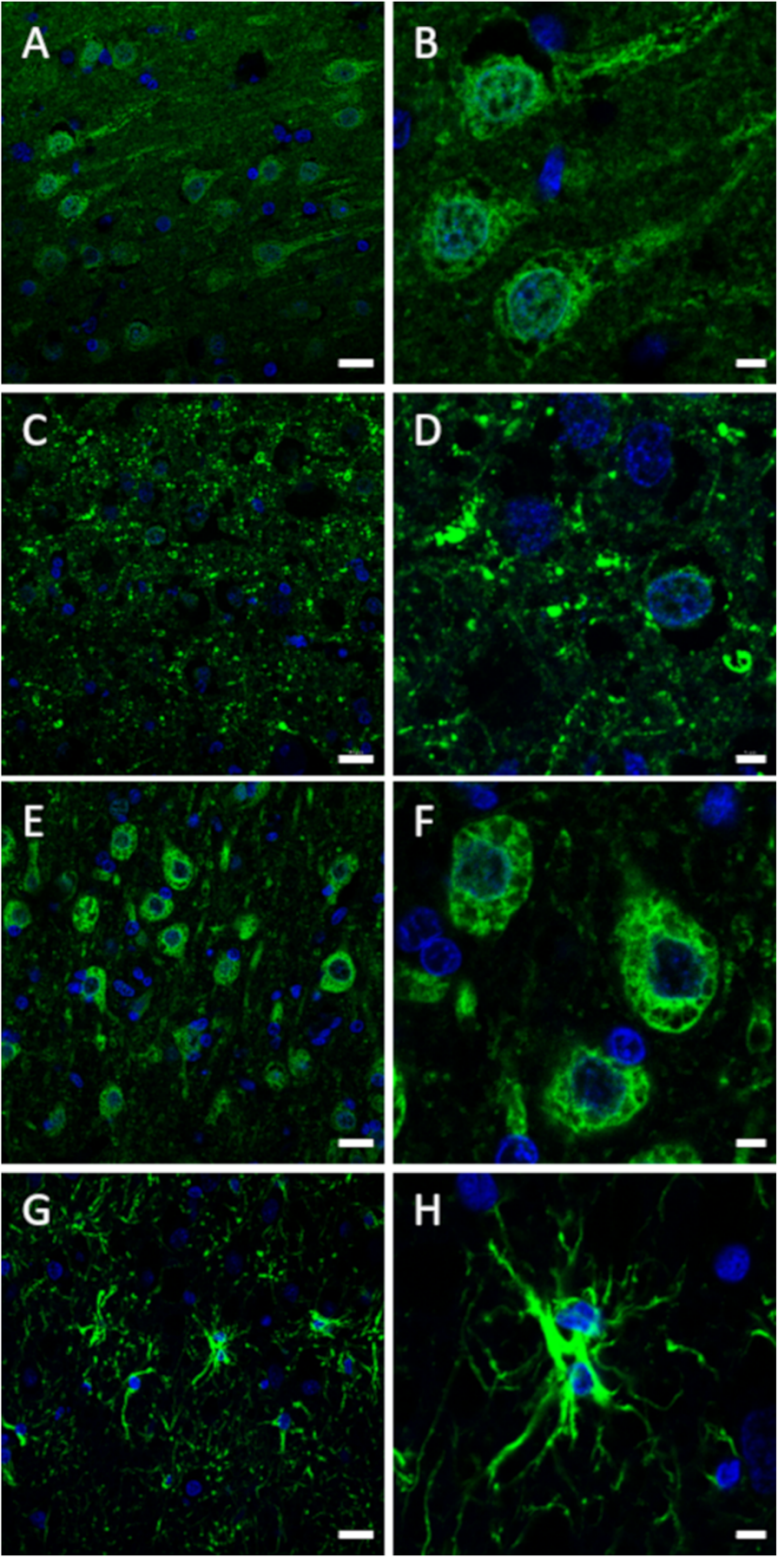
Canine brain immunofluorescence stainings. The frontal cortex of a 16-year-old dog was stained with antibodies against TUBB3 (**a**, **b**), NF-H (**c**, **d**), MAP2 (**e**, **f**), and GFAP (**g**, **h**). Nuclei were counterstained with DAPI (blue). Scale bars are 20 µm in images in the left column and 5 µm in images in the right column

Canine brain tissue sections were stained with antibodies directed against neural markers to determine the reactivity of these antibodies against canine epitopes and thus the usefulness for their further use on canine cell cultures. Neurons labelled positively with antibodies directed against NF-H, MAP2, and TUBB3, and astrocytes labelled positively with antibodies against GFAP (Fig. 5). Antibodies directed against nestin did not detect nestin in the canine brain.

**Fig. 6:**
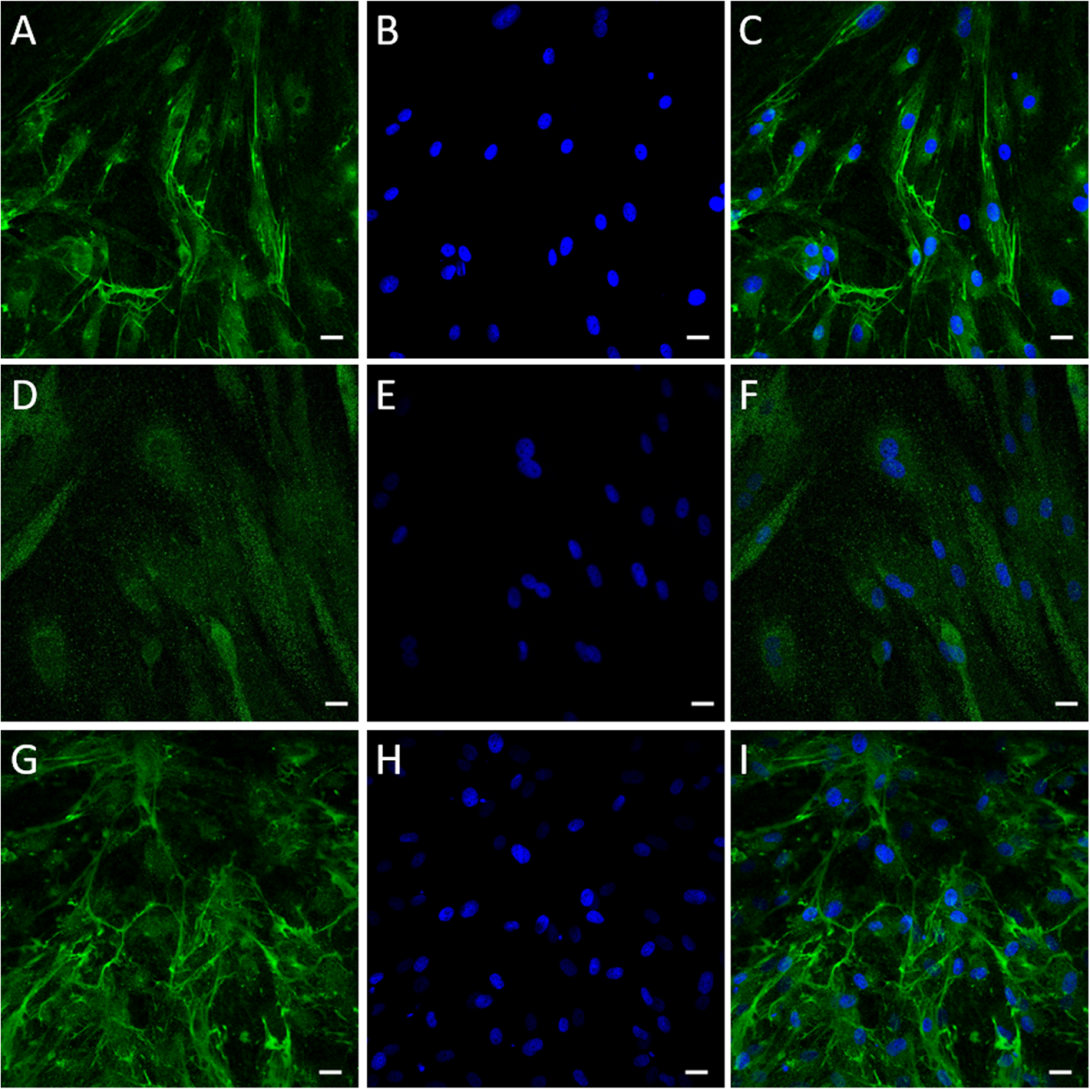
Expression of neural markers TUBB3 (**a**, **c**), NF-H (**d**, **f**), and GFAP (**g, i**) in canine ASCs exposed to differentiation medium NIMa for three days. Prior to the addition of NIMa, cells were grown in pre-differentiation medium STIM1 for 24 h. Nuclei were counterstained with DAPI (blue). Scale bars are 20 µm

**Fig. 7:**
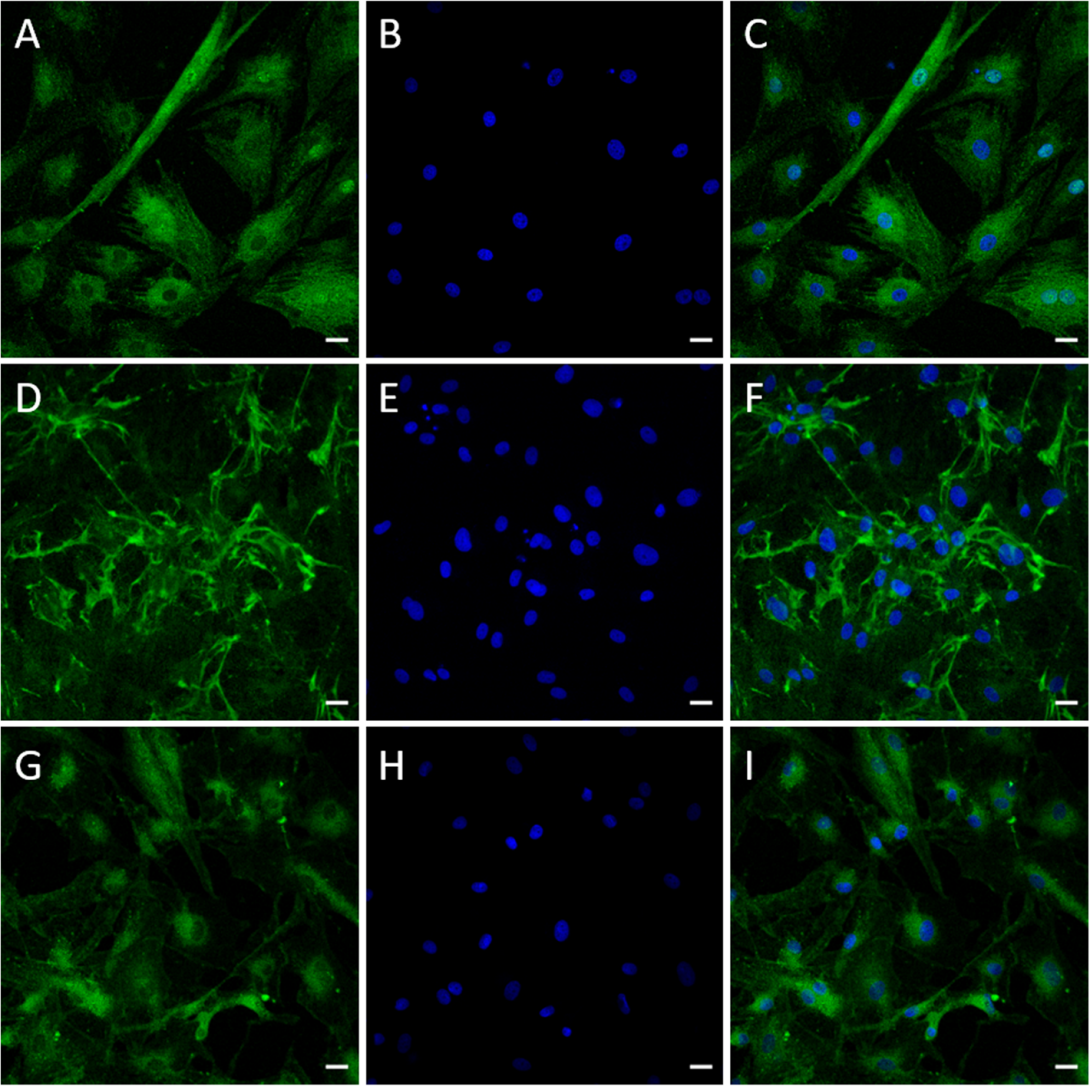
Expression of neural markers TUBB3 (**a**, **c**), NF-H (**d**, **f**), and GFAP (**g, i**) in canine ASCs exposed to differentiation medium NIMa for nine days. Prior to the addition of NIMa, cells were grown in pre-differentiation medium STIM1 for 24 h. Nuclei were counterstained with DAPI (blue). Scale bars are 20 µm

The differentiation of canine ASCs into neural lineages was determined by the expression of TUBB3, NF-H, and GFAP. Immunocytofluorescence analysis revealed the presence of the neuronal cytoskeleton proteins TUBB3, NF-H, and GFAP, a marker of glia cells, after 3 and 9 days of growth in the differentiation medium NIMa (Figs. 6 and 7).

### Flow cytometry

**Fig. 8:**
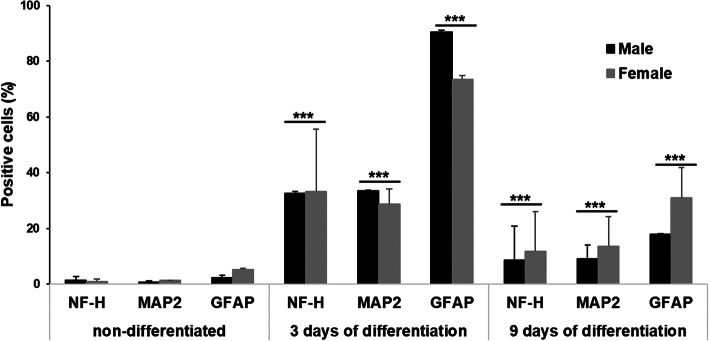
Percentage of NF-H, MAP2, and GFAP positive cells before the differentiation and 3 or 9 days after differentiation in NIMa medium. Flow cytometry analysis revealed significant difference in expression of all three markers between undifferentiated cells and cells after 3 and 9 days of differentiation (^***^ *p* < 0.001). Data are presented separately for female and male derived ASCs as mean percentages ± SD. Differences between sexes were not statistically significant

The expression of neural markers NF-H, MAP2, and GFAP was further characterized by flow cytometry in ASCs grown in differentiation media for 3 and 9 days. As the size of multipotent mesenchymal stromal cells is similar to the size of lymphocytes, the gating of cells was done similarly. The expression of markers was comparable for differentiated cells derived from female and male dogs (Fig. 8). The average expression of NF-H in undifferentiated cells from male donors was 1.4 ± 0.8%, MAP2 0.7 ± 0.4%, and GFAP 2.3 ± 1.4% and from female donors NF-H 0.9 ± 0.3%, MAP2 1.4%, GFAP 5.3 ± 1.1%. After 3 days of differentiation in NIMa media, the expression of all three markers significantly increased (p < 0.001 for all markers) in cells from both male and female dogs, with no difference between sexes. The average expression in cells from male dogs was: NF-H 32.6 ± 0.8%, MAP2 33.6 ± 0.1%, GFAP 90.4 ± 0.6% and in ASCs from female dogs: NF-H 33.3 ± 1.4%, MAP 22.8 ± 5.4%, GFAP 73.5 ± 22.3%. The expression of all markers decreased 6 days later (after 9 days of differentiation) with cells derived from male dogs to NF-H 8.7 ± 0.4%, MAP2 9.0 ± 5%, GFAP 17.8 ± 12.1% and in cells derived from female dogs to NF-H 11.7 ± 11.0%, MAP2 13.6 ± 10.7%, GFAP 30.9 ± 14.3%, but the expression of all three markers was still significantly different from expression in undifferentiated cells (*p* < 0.001; Fig. 8). The percentage of differentiated cells after 9 days of differentiation was lower in male than in female dogs, but the difference was not statistically significant.

## Discussion

The differentiation of MSCs into neural cells makes them interesting and potentially useful for neural reconstitution in neurodegenerative diseases, stroke, and spinal cord injuries. However, it is not known whether the transplantation of ASCs alone would be sufficient to treat spinal cord injuries and other neurological disorders or *in vitro* differentiation would be needed before the transplantation. Therefore, there is a need to develop optimal procedures for the induction of neuronal differentiation of MSCs.

In the present study, we confirmed that canine adipose tissue-derived MSCs are capable of neural differentiation *in vitro* and, furthermore, explored which neural induction medium is the most suitable for the neural differentiation of canine ASCs. In previous studies, rat and human multipotent mesenchymal stromal cells were shown to transdifferentiate into neural phenotypes by exposing these cells to a variety of neurogenic inductors, such as β-mercaptoethanol, butylated hydroxyanisole, potassium chloride (KCl), valproic acid, and forskolin [2, 5, 8, 11, 12]. Alternative methods to the chemical differentiation of human and canine MSCs into a neural lineage involves the addition of growth factors such as bFGF, EGF, neuroblast factor (N2), B27 supplement, and retinoic acid [7, 13, 15, 22]. One study also showed that canine adipose tissue-derived stromal cells could be differentiated into neuronal cells by incubation in the presence of dibutyryl cyclic adenosine monophosphate (dbcAMP) and isobuthylmethylxanthine (IBMX) [17].

We tested two pre-differentiation media to condition the cells to neural differentiation. Serum-free medium (STIM1) with added growth factors EGF, bFGF, and B27, supplement was suitable for cell culture whereas STIM2 turned out to be highly toxic to the cells, probably due to the high concentration of β-mercaptoethanol. This pre-differentiation step was introduced following previous studies which show that culturing ASCs under active proliferation conditions greatly improves their propensity to differentiate toward neurogenic lineages [23]. The neurogenic inductors in differentiation media tested were B27 supplement, valproic acid, forskolin, KCl, and butylated hydroxyanisole (BHA) in the KEM medium and B27 supplement, N2 supplement, and retinoic acid in NIM media. As KEM was toxic to the cells and cells died after only 3 days of incubation, the NIM media were further assessed for their ability to trigger the neural differentiation of canine ASCs. In the NIMa medium, which contained the lowest concentration of retinoic acid (10 nM), the most prominent neural-like cellular phenotypes were observed; thus, this was the medium of choice for subsequent experiments. Retinoic acid, a metabolite of vitamin A, has roles in cell differentiation, neurite outgrowth and cell survival [24, 25]. It induces post-mitotic neuronal phenotypes in various cells *in vitro* [24, 26, 27] and might have been the crucial co-factor in NIMa medium for canine ASCs to switch from proliferation to differentiation. Although ASCs seem to differentiate into neural phenotypes also in media NIMb and NIMc, there were many apoptotic cells in the cell culture with these two media. Retinoic acid could be highly toxic to cells both *in vitro* and *in vivo*, and higher concentrations of retinoic acid in media NIMb and NIMc was probably causing cell deaths in these two media.

Cell numbers were the highest on the third day after the addition of the NIMa medium; cells from male and female dogs grew similarly during the neural induction. The viability of all cells was reduced on day 9 of differentiation and was statistically significantly lower in ASCs derived from female than male donors. Stressors, such as neurogenic factors present in NIMa medium and/or low serum concentration and poor nutrition, might have increased the ASCs death rate. Why female cells were more sensitive to these effects remains unexplained and will have to be studied in the future. One option that might be interesting to test in the future studies would be the addition of estradiol to the differentiated cells. Estradiol has many significant functions in various parts of the brain and perhaps adult female cells need estradiol for their optimal survival.

Cell population obtained by the cultivation of ASCs in neural-differentiation medium is often a mixture of cells expressing one or more neural-specific markers. The most studied markers included nestin, TUBB3, S100, and GFAP [28]. Differentiated rat and human multipotent mesenchymal stromal cells were shown to express mature neural markers, such as GFAP, MAP2, TUBB3, and neuron-specific enolase (NSE). Such cells also possess voltage-gated calcium channels and the ability to upregulate the glutamate receptor [2, 5, 8, 11, 12]. In the present study, after 24-hour preconditioning in pre-differentiation medium STIM1 and subsequent incubation in NIMa differentiation medium for 3 or 9 days, cells expressed proteins characteristic for mature neurons and astrocytes. Immunofluorescence analysis showed the expression of the neuronal markers NF-H and TUBB3, and the glial marker GFAP. The neural phenotype was confirmed by flow cytometry showing very high expression of GFAP after 3 days of growth in the differentiation medium, and an increase of mature neuronal markers NF-H and MAP2 expressing cells in comparison to undifferentiated cells. In undifferentiated cells, the basal expression of neural markers was also detected but at very low levels. This basal level of expression of neural markers in a cell culture of undifferentiated canine ASCs was reported in a recent study, in which some of the neural markers and neurotrophic factors were expressed in undifferentiated cells, albeit at low levels [14].

The expression of neural markers in our study was the highest after three days of growing in the differentiation medium. After 9 days of cell culture, the expression of all markers tested was reduced. This reduction in immunopositive cells might have been due to the de-differentiation of cells or apoptosis of differentiated cells. None of the previous studies on neural differentiation of canine ASCs followed the expression of neural markers *in vitro* for more than 3 days; it thus remains unknown if the drop in the expression might have occurred in other studies if the cells had remained in an induction media for longer periods.

MSCs directed at least partially to neuronal lineages could provide an opportunity in developing novel treatments for a variety of disorders of the central nervous system such as spinal cord injuries and neurologic conditions affecting the brain (Alzheimer’s, Parkinson’s, Huntington’s disease, stroke, cerebral palsy, brain ischemia, traumatic brain injury, amyotrophic lateral sclerosis, etc.). As dogs and humans share their environment and have similar lifestyles, they also suffer from some similar diseases. For instance, canine cognitive dysfunction is in many aspects similar to human Alzheimer’s disease [29] and represent an interesting model to study this debilitating disease. Canine cognitive dysfunction is interesting disease for cell replacement therapy by transplanting neurally differentiated ASCs into the canine brain. A previous study has shown that human umbilical cord-derived MSCs, transplanted into canine brains, migrated and enhanced endogenous neural stem cell population in the subventricular zone [30]. This indicates that MSCs secreted some factors to support the neural differentiation of endogenous stem cells. This has also been corroborated in a Phase 1 human clinical trial of AD, in which repeated intracerebroventricular injections of adipose-derived stromal vascular fraction improved cognition in AD patients [31]. The therapy with partially neurally differentiated autologous MSCs would be potentially even more beneficial, as it could exploit the neuroprotective and neurotrophic features of MSCs and the neurally differentiated cells would provide the supportive (glia) and functional (neurons) roles to the diseased/injured brain. In this way, various neurological conditions could be potentially treated in dogs, since the autologous ASCs are easily accessible, and their expansion, differentiation, and subsequent transplantation could be strictly monitored. However, for potential future use in regenerative medicine, the timing of neuronal differentiation would have to be explored meticulously, as this could be one of the critical points to achieve the highest quality of cells used for treatments.

## Conclusions

The differentiation repertoire of canine ASCs clearly extends beyond mesodermal lineages. *In vitro* induction of ASC into neural lineages could be exploited in the future to treat neurological conditions. ASCs already possess neuroprotective properties such as anti-inflammatory and anti-astrogliosis effects, and neuronal induction could supplement these functions by enabling neuronal regeneration. However, as results of this study show, there are significant differences in the ability of different media to induce neural differentiation; therefore, in future studies, the optimal composition of growth media will have to be determined, optimized, and synchronized in order to develop general guidelines for the induction of canine ASCs into neural cells.

## Methods

### Cell origin

Adipose tissue was collected by veterinarians at veterinary clinics. All dogs were privately owned patients and were under general anaesthesia for other procedures, not purposefully to collect samples for this study. All the dogs’ owners gave written consent to use the removed adipose tissue for research purposes, and all the experiments were performed according to the procedures and guidelines approved by the National Health Service branch of the Slovenian Ministry of Health. Additional ethical permission was not needed according to Slovenian legislation and interpretation by Administration for Safe Food, Veterinary and Plant protection, which is responsible for issuing licenses for experiments with animals. Subcutaneous adipose tissue (< 1 cm³) was aseptically removed from the back area between the dog’s scapulae. Samples were obtained from six different dogs aged 2 to 9 years (mean age 5.5 years) of different breeds and both sexes, three males and three females. The inclusion criteria were that all the canine donors were healthy, and the ASCs were in the early passages.

### Isolation of canine ASCs

Canine ASCs were isolated from adipose tissue and characterized based on the routine protocols developed by Animacel Ltd. Briefly, fat tissue was minced, washed three times in PBS buffer and digested over night with an equivalent amount of collagenase I solution containing 2 mg/ml collagenase I and 4 mg/ml BSA in HEPES buffer, pH 7.4. Collagenase I activity was stopped by a double volume of PBS and the resulting cell suspension was filtered through a falcon strainer (pore size 100 µm). Cells were grown in the cultivation medium, which was previously demonstrated to be specific for canine ASCs cultivation [32] and was developed by Animacel Ltd. After 24 h, non-adherent cells were washed away. ASCs were cultured until 80% confluence was reached, detached, counted with Bürker Turk Chamber hemocytometer and passaged. Again, cells were maintained until 80% confluence, detached and cryopreserved in a freezing medium consisting of 70% DMEM Glutamax, 20% FBS, and 10% DMSO, for later use.

### Cultivation of ASCs

The high cell density is a key parameter for the successful differentiation of multipotent mesenchymal stromal cells into neural lineages. Isolated ASCs (first passage) were plated into different growth flasks depending on the designed experiment. During the optimization and monitoring of the morphology of undifferentiated and differentiated cells, two T25 flasks were used for cells from each dog, plated at a density of 10^6^ cells/cm². Three additional T25 flasks were required for flow cytometry, for each point of differentiation, and for each animal, with the cells at the same density. Furthermore, for the immunocytochemical characterization of differentiated ASCs, individual 35 mm petri dishes were used with cells at a density of 8 × 10^4^ cells/cm² and 24-well plates with cells plated on glass coverslips coated with laminin at a density of 2 × 10^4^ cells/cm².

### Neural differentiation

Canine ASCs in a second passage were plated in different flasks based on the type of the experiment. Cells were grown in our standard cultivation medium. After 24 h, the cultivation medium was replaced by the pre-differentiation neural induction medium (mitogenically stimulated; STIM). Two pre-differentiation media were tested. STIM1 consisting of DMEM Glutamax, EGF (20 ng/ml), bFGF (20 ng/ml), and 1x B27 supplement (supplied as 50 × stock solution, Gibco); and STIM2 consisting of DMEM Glutamax, β-mercaptoethanol (100 µM), and 20% FBS. After 24 h, the pre-differentiation medium was replaced by the neural differentiation medium. Four different differentiation media were prepared:

1. KEM media consisting of DMEM Glutamax, 1% FBS, 2 × B27 supplement, valproic acid (2 mM), forskolin (10 µM), hydrocortisone (1 µM), KCl (5 mM), butylated hydroxyanisole (BHA; 200 µM), insulin (5 µg/ml), and 0.1% penicillin/streptomycin.
2. NIMa (neural induction medium a) composed of DMEM Glutamax, 2 × B27 supplement, 1 × N2-supplement, 1% FBS, 0.1% penicillin/streptomycin, and 10 nM retinoic acid.
3. NIMb consisting of DMEM Glutamax, 2 × B27 supplement, 1 × N2-supplement, 1% FBS, 0.1% penicillin/streptomycin, and 100 nM retinoic acid.
4. NIMc composed of DMEM Glutamax, 2 × B27 supplement, 1 × N2-supplement, 1% FBS, 0.1% penicillin/streptomycin, and 10 µM retinoic acid.

The cells were further processed for morphological, immunocytochemical and flow cytometry analysis after 3, 6, and 9 days following neural induction.

### Viability assay

To determine the cell numbers and the viability of canine ASCs cells after exposure to neural differential medium, a dye exclusion assay was used. The viability was determined at three time points (after 24 h of pre-differentiation in STIM1 medium and at 3 and 9 days after induction of differentiation) by adding Trypan Blue dye into the cell suspension and counting live/dead cells by using a Bürker Turk Chamber under the light microscope. The viability of cells grown in control media was determined for comparison to neurally differentiated cells. All experiments were performed in triplicate.

### Immunohistochemistry on dog brain

Dog brain tissue sections were used as a control for the reactivity of antibodies. A dog’s brain was obtained at Veterinary Faculty of the University of Ljubljana following the dog owner’s approval. The dog was a sixteen-year-old male, euthanized due to advanced cognitive dysfunction. Prior to euthanasia the dog was sedated with propofol (0.5 mL per kg) and then euthanised with T61 (a mixture of embutramide, mebezonium iodine, and tetracaine hydrochloride). The brain was surgically removed, cut into small cubes and fixed in 4% paraformaldehyde at 4 °C for several days. Pieces of the brain were embedded in paraffin using an automated tissue processor (Tissue processor Leica TP 1020). Tissue blocks containing the area of the frontal cortex were cut to 7 µm thick sections and further processed for immunohistochemistry. After dewaxing, sections were subjected to antigen retrieval in sodium citrate buffer (10 mM sodium citrate, 0,05% Tween 20, pH 6) by boiling the slides for 10 min in a microwave oven; this was followed by blocking the unspecific epitopes in 1.5% BSA, 10% normal goat serum, 0.1% TritonX-100 in PBS for 60 min at room temperature. Sections were then incubated overnight at 4 °C with the following primary antibodies: mouse anti-GFAP (1:400, G3893, Sigma), mouse anti-NF-H (1:400, AB1989, Millipore), mouse anti-bIII tubulin (1:400, sc-80,005, Santa Cruz Biotechnology), rabbit anti-MAP2 (1:400, AB5622, Millipore), and chicken anti-nestin (1:200, ABIN187958, Neuromics Antibodies). The next day slides were washed with PBS for 5 min and incubated with secondary antibodies (anti-rabbit Alexa Flour 555 or anti-mouse Alexa Flour 555 diluted 1:1000 in blocking buffer (both from Invitrogen)) for 1 h at room temperature in the dark. The nuclei were counterstained with DAPI (Sigma). Slides were then washed with PBS thrice for 5 min and mounted with ProLong Gold Antifade Mountant (Molecular Probes). Stainings were visualized with a confocal microscope (Zeiss LSM 710) and ZEN software.

### Immunofluorescence analysis of cells

Immunocytochemistry was performed on the cells to evaluate the presence of the neural markers after the induction with differentiation medium. One 24-well plate was prepared per each time point with round glass coverslips coated with laminin (10 µg/ml). The next day, the second passage canine ASCs were plated at a density of 2 × 10^4^ cells/cm² in 1 ml of the basal growth medium. At 3 and 9 days of differentiation cells were fixed with 4% paraformaldehyde (pH 7.4) for 10 min at room temperature, rinsed three times in PBS and permeabilized with TBST for 5 min. The blocking of unspecific epitopes was performed with blocking solution (10% FBS, 1% milk powder, 0.02% Na-azide, TBST, pH 7,2) for 1 h. Cells were then incubated overnight at 4 °C with the primary antibodies: rabbit anti-GFAP (1:400, G3893, Sigma) conjugated with Alexa Flour 488, rabbit-anti-NF-H (1:400, AB1989, Millipore), and rabbit anti-bIII tubulin (1:1000, AB9354, Millipore). The next day, the cells were rinsed three times with PBS and one time with 2% FBS in PBS and incubated with secondary donkey anti-rabbit Cy2 conjugated antibody or secondary donkey anti-mouse Cy3 conjugated antibody (both 1:500; Jackson Immunoresearch) for 2 h at room temperature in dark. Anti-GFAP antibody was covalently labelled with mouse anti-rabbit Alexa Flour 488 IgG using Alexa Fluor® 488 Protein Labeling Kit (Molecular Probes). The nuclei were counterstained with DAPI. Stainings were visualized with a confocal microscope (Zeiss LSM 710). Neural differentiation was detected by observing random viewing fields under the microscope and by comparing cellular morphology. NF-H and TUBB3 positive cells were identified as neuronal lineage, and GFAP-positive cells were identified as glial lineage (astrocytes).

### Flow cytometry

ASCs cell cultures were detached for flow cytometry analyses at three time points (pre-differentiation, 3, and 9 days of differentiation). Confluent cells were harvested using trypsin (TrypLE™ Express, Gibco) digestion for 5 min at 37 °C, centrifuged for 5 min at 1400 rpm at 4 °C and resuspended at a concentration of 10^7^ cells/ml in PBS. Five aliquots were prepared per time point (pre-differentiation, 3, and 9 days of differentiation) per animal each containing 100 µl (concentration of 10^6^ cells/ml) and were transferred to flow cytometry facility at the Medical Faculty, University of Ljubljana. The aliquots were centrifuged for 5 min at 1600 rpm at room temperature, and pellets were fixed with 4% paraformaldehyde (pH 7.4) for 10 min at room temperature, rinsed three times in PBS, permeabilized with 0.1% Triton X-100, and blocked with 2% FBS and 5% milk powder for 10 min. Cells were incubated for 30 min in the dark at 37 °C with following primary antibodies: rabbit anti-NF-H (1:400, AB1989, Millipore), rabbit anti-MAP2 (1:200, AB5622, Millipore), and mouse anti-GFAP (1:400, G3893, Sigma) conjugated with Alexa Flour 488 (Life Technologies). After incubation, samples were centrifuged 5 min at 1600 rpm, rinsed two times with 2% FBS in PBS and the ones stained for NF-H and MAP2 incubated with secondary donkey anti-rabbit IgG Cy2 conjugated antibody (1:500, Jackson Immunoresearch). Anti-GFAP antibody was previously labelled as described above. All samples were analysed with a BD FACS Canto flow cytometer (BD Biosciences). Appropriate isotype-matched controls conjugated to FITC were used to identify nonspecific staining for GFAP antibody. For unconjugated primary antibodies, controls included isotype-matched unconjugated primary controls and incubation with the secondary antibody alone. Data were analysed using FACSDiva™ version 6.1.2 (BD Biosciences) analysis software.

### Statistical analysis

Immunocytochemical staining and cell morphology were evaluated only qualitatively by observing random viewing fields by observer blind to the differentiation conditions. Differences in cell numbers and cell viability between control and differentiated ASCs were analysed with repeated measures ANOVA followed by a Bonferroni post-hoc test with sex as an independent variable and day of culture as a within factor. Differences between groups in the expression of neuronal markers detected by flow cytometry were analysed with ANOVA followed by a Bonferroni post-hoc test. All differences were considered statistically significant at *P* < 0.05.

## Data Availability

The datasets used and/or analysed during the current study are available from the corresponding author on reasonable request.

## Abbreviations

ASCs: Adipose tissue-derived multipotent mesenchymal stromal cells/mesenchymal stem cells
BDNF: Brain-derived neurotrophic factor
bFGF: Basic fibroblast growth factor
EGF: Epidermal growth factor
FBS: Foetal bovine serum
GFAP: Glial fibrillary acidic protein
MAP2: Microtubule-associated protein 2
MSCs: Multipotent mesenchymal stromal cells
NGF: Nerve growth factor
NIMa: Neural induction medium a
NIMb: Neural induction medium b
NIMc: Neural induction medium c
NF-H: Neurofilament H
TUBB3: Tubulin beta III
